# Extracellular Vesicles Are Key Regulators of Tumor Neovasculature

**DOI:** 10.3389/fcell.2020.611039

**Published:** 2020-12-09

**Authors:** Naoya Kuriyama, Yusuke Yoshioka, Shinsuke Kikuchi, Nobuyoshi Azuma, Takahiro Ochiya

**Affiliations:** ^1^Division of Molecular and Cellular Medicine, Institute of Medical Science, Tokyo Medical University, Tokyo, Japan; ^2^Department of Vascular Surgery, Asahikawa Medical University, Asahikawa, Japan

**Keywords:** extracellular vesicles, cancer, tumor microenvironment, tumor metastasis, tumor angiogenesis

## Abstract

Tumor progression involves a series of biologically important steps in which the crosstalk between cancer cells and the surrounding environment is an important issue. Angiogenesis is a key tumorigenic phenomenon for cancer progression. Tumor-related extracellular vesicles (EVs) modulate the tumor microenvironment (TME) through cell-to-cell communication. Tumor cells in a hypoxic TME release more EVs than cells in a normoxic environment due to uncontrollable tumor proliferation. Tumor-derived EVs in the TME influence endothelial cells (ECs), which then play multiple roles, contributing to tumor angiogenesis, loss of the endothelial vascular barrier by binding to ECs, and subsequent endothelial-to-mesenchymal transition. In contrast, they also indirectly induce tumor angiogenesis through the phenotype switching of various cells into cancer-associated fibroblasts, the activation of tumor-associated ECs and platelets, and remodeling of the extracellular matrix. Here, we review current knowledge regarding the involvement of EVs in tumor vascular-related cancer progression.

## Introduction

Extracellular vesicles (EVs) were first discovered in 1967 and initially called “platelet dust,” which was defined as subcellular coagulant materials (Wolf, 1967; Cufaro et al., 2019). Thereafter, platelet dust has been called microparticles (MPs) or microvesicles (MVs) in general. However, accumulating evidence for mechanisms associated with EVs indicate that EVs are involved in various cell-to-cell signaling pathways and act as important molecular messengers in various pathophysiological processes (Cufaro et al., 2019). EVs are released from almost all cells and phospholipid bilayer membranous vesicles (Thery et al., 2018). According to the International Society for Extracellular Vesicles (ISEV), EVs are categorized into several subtypes according to their size or biogenesis: exosomes, MVs (microvesicles, ectosomes, or microparticles), and apoptotic bodies (Witwer et al., 2013; Cufaro et al., 2019). Exosomes are believed to originate from intraluminal vesicles by merging intracellular multivesicular bodies (MVBs) with the plasma membrane (Witwer et al., 2013; Cufaro et al., 2019). MVBs contain vesicles and combine with lysosomes for degradation of their contents or combine with the plasma membrane, resulting in the effusion of intraluminal vesicles, defined as exosomes, into the extracellular space (Harding et al., 1983; Pan et al., 1985). Exosomes are approximately 100 nm in diameter and smaller than MVs (Witwer et al., 2013). The membranous proteins of exosomes are enriched with heat shock proteins (HSP70, HSP90), integrins, Alix, tetraspanins (CD9, CD63, CD81, CD82, and CD151), MHC class II proteins, epithelial cell adhesion molecules, and members of the human epidermal receptor family (Zhang and Grizzle, 2014; Kalluri, 2016). In contrast, MVs are a few nm to a few μm in diameter and originate from outward budding from the plasma membrane (Witwer et al., 2013; Kalluri, 2016; Tkach and Théry, 2016). Apoptotic bodies are released by apoptotic cells; they are 1–5 μm in diameter and the largest EVs (van der Pol et al., 2012; Cufaro et al., 2019). However, there are currently no indubitable markers to specifically identify these vesicles. Thus, the ISEV consensus recommends using the generic term EV in the nomenclature (Gould and Raposo, 2013). EVs contain various types of molecules, such as proteins, lipids, carbohydrates, DNAs, and coding and non-coding RNAs (Nawaz et al., 2018). EVs have various types of functions and can mediate cell-to-cell communication. They are taken up by various cells via autocrine, paracrine, and endocrine processes (Zhang and Grizzle, 2014). EVs are involved in various physiological processes, such as atherosclerosis (Hafiane and Daskalopoulou, 2018; Deng et al., 2019), neurodegenerative diseases (Grad et al., 2014), autoimmune diseases (Beyer and Pisetsky, 2010; Willis et al., 2019; Wu et al., 2019), and cancer progression (Cufaro et al., 2019).

It is clear that the majority of cancer-related mortalities are the result of cancer metastasis (Eccles and Welch, 2007). Cancer metastasis is a complex process that includes detachment from the primary tumor and invasion into adjacent tissues, evasion from the immune system, transport into the circulation, and extravasation and growth at a secondary organ (Mehlen and Puisieux, 2006; Li et al., 2019). The tumor microenvironment (TME) is the environment around the tumor and plays important roles in cancer progression and metastasis (Guo and Deng, 2018). The TME consists is highly organized and includes blood vessels, lymph vessels, endothelial cells (ECs), pericytes, fibroblasts, macrophages, and lymphocytes (Balkwill et al., 2012; Baghban et al., 2020). The TME also includes non-cellular components. For example, the extracellular matrix (ECM) is one of the important components of the TME and includes collagen, fibronectin, hyaluronic acid, laminin and other molecules (Baghban et al., 2020). In addition to these molecules, studies on the TME have also focused on EVs in. Numerous studies have suggested that tumor cells and stromal cells in the TME release a large number of EVs that mediate cell-to-cell communication and that the surrounding microenvironment plays essential roles in cancer metastasis (Tominaga et al., 2015a; Kalluri, 2016; Li et al., 2019).

The TME is more hypoxic than the normal internal environment due to the uncontrollable growth of cancer cells (Jing et al., 2019; Tan et al., 2020). Solid tumors, which require nutrients and oxygen under hypoxic conditions, develop vessels that supply the tumor (Vaupel and Harrison, 2004; Jing et al., 2019). Cancer-related neovessels, such as those observed during angiogenesis and lymphangiogenesis, are needed for invasive tumor growth and metastasis. Angiogenesis refers to the growth of new blood vessels and is one of the most important processes for invasive tumor growth and cancer metastasis. Tumor angiogenesis supplies oxygen and nutrients to not only cancer cells but also the premetastatic niche, and the primary tumor prepares the microenvironment for distant organs to promote cancer metastasis, angiogenesis and vascular permeability (Liu and Cao, 2016). Tumor angiogenesis is also associated with various types of cells, such as tumor cells, ECs, pericytes, lymphocytes, monocytes/macrophages, fibroblasts, and platelets (De Palma et al., 2017). According to recent studies, EVs secreted by these cells are substantially involved in cancer neovasculature and metastasis. In this review article, we summarize recent findings on the relationship between cancer-related EVs and the TME, tumor-related vasculature, and metastasis.

## Functional EVs Released by Tumors Under Hypoxia

Solid tumor cells near functional blood vessels, in which the normoxic area has an oxygen level of 38 mmHg (5%) (McKeown, 2014), are viable and proliferative. However, as a result of growing solid tumors at a distance of approximately 200 μm from normal functional vessels, tumor cells are exposed to a hypoxic environment induced by an inadequate oxygen supply due to an increase in diffusion distance (Wilson and Hay, 2011). Hypoxia due to this uncontrollable proliferation of tumor cells results in an oxygen level below 10 mmHg (1.3%) (McKeown, 2014). This hypoxic environment may become anoxic and give rise to cell necrosis. Furthermore, a tumor mass exceeding 1–2 mm in diameter without a vascular supply can prevent cancer cell proliferation (Mao et al., 2019). Genomic changes in tumor cells induced by a hypoxic environment lead to the adaptation of tumor cells to poor nutrition and a poor oxygen supply (Al Tameemi et al., 2019). Tumor cells lead to the formation of new blood vessels that bring nutrients and oxygen to survive and overcome proliferation limitations in a hypoxic microenvironment. Hypoxia is one of the first steps of cancer metastasis, one of the most intensively studied characteristics of the TME (Meng et al., 2019), and is strongly associated with immune evasion, the evasion of apoptosis, tumor growth, angiogenesis, and metastasis (Walsh et al., 2014; Al Tameemi et al., 2019).

**FIGURE 1:**
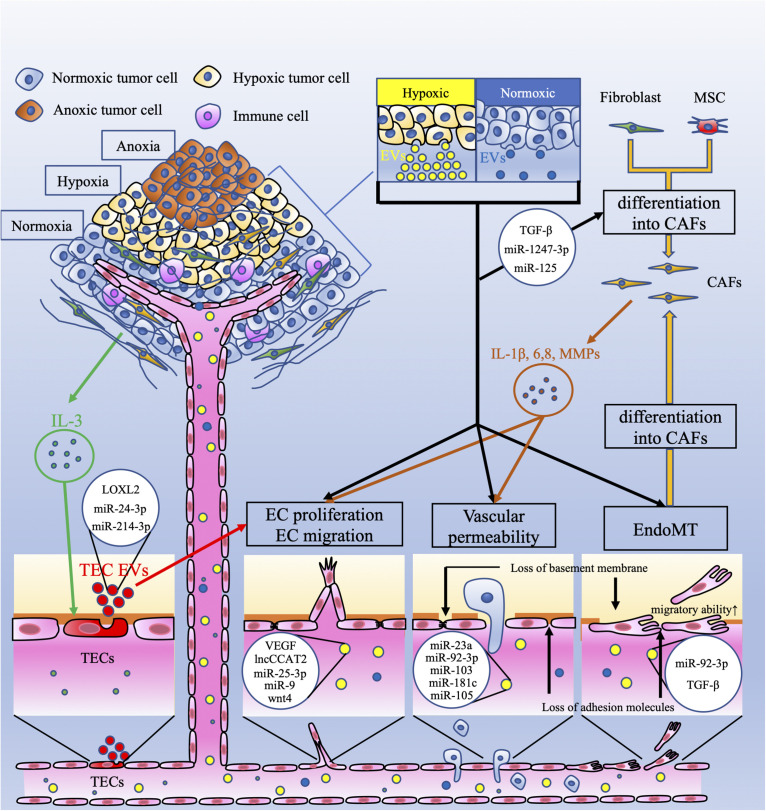
Crosstalk between tumor cells and endothelial cells via extracellular vesicles. This figure shows the vascular-related tumor microenvironment (TME) and extracellular vesicles (EVs). Tumor cells under hypoxia produce more extracellular vesicles (EVs) than those under normoxia. These EVs have several effects on endothelial cells (ECs). EC proliferation and migration are induced by EV-related vascular endothelial growth factor (VEGF), the long non-coding (lnc) RNA CCAT2, miR-25-3p, miR-9, and wnt4. Vascular permeability is increased by EV-related miR-23a, miR-92-3p, miR-103, miR-181c, and miR-105. Interleukin 3 (IL-3), secreted by tumor cells, activates ECs (TECs), which secrete EVs and promote neovessel formation. These EVs contain lysyl oxidase like-2 (LOXL2), miR-24-3p, and miR-214-3p. EVs derived from tumor cells, which contain miR-92-3p and/or TGF-β, downregulate endothelial characteristics and acquire mesenchymal characteristics in ECs; this is referred to as endothelial-mesenchymal transition (EndoMT). EndoMT is also induced by tumor-derived EVs. EndoMT can be the source of cancer-associated fibroblasts (CAFs). Fibroblasts and mesenchymal stem cells (MSCs) can also differentiate into CAFs by tumor-derived EVs. CAFs secrete interleukin-1β (IL-1β), IL-6, IL-8, and matrix metalloproteinases (MMPs). These factors induce EC proliferation and migration and increase vascular permeability. These mechanisms mediated by EVs contribute to tumor angiogenesis and metastasis.

Tumor cells in a hypoxic environment produce more EVs than cells in a normoxic environment, and this environment substantially alters the protein and nucleic acid profiles of EVs (King et al., 2012; Wang T. et al., 2014; Figure 1). EVs induced by hypoxia may have a strong effect on cancer metastasis and angiogenesis. Tumor-derived EVs and stromal cell-derived EVs under hypoxic conditions can mediate crosstalk between each other (Meng et al., 2019). For example, hypoxia-related EVs increase the expression of miR-210, which promotes tumor progression through EC tubulogenesis (Fasanaro et al., 2008; Jung et al., 2016). In ovarian cancer, hypoxic conditions induce STAT3, which downregulates Rab7 and upregulates Rab27, resulting in an increase in EV release (Dorayappan et al., 2018). The EV-associated miR-135b derived from hypoxia-resistant multiple myeloma cells suppresses the expression of factor inhibiting hypoxia-inducible factor 1 (FIH-1), a negative regulator of the transcriptional activity of HIF-1α that promotes endothelial tube formation (Umezu et al., 2014). These studies suggest that tumor cell-derived EVs, which increase under hypoxia, can directly enhance tumor angiogenesis.

Several studies have shown that tumor cells release more EVs under hypoxic conditions than under normoxic conditions in a hypoxia inducible factor (HIF)-dependent manner. HIFs have various effects on cellular functions and control various types of oxygen responsive target genes. HIF-1α induces various angiogenetic growth factors, including vascular endothelial growth factor (VEGF), stromal derived factor 1 (SDF1), angiopoietin 2, placental growth factor, platelet derived growth factor (PDGF), and stem cell factor (Rey and Semenza, 2010). These factors stimulate ECs, pericytes, and vascular smooth muscle cells and promote the formation of new capillary vessels (Rey and Semenza, 2010).

The release of MVs or EVs is promoted by the HIF pathway. Dimethyloxalylglycine (DMOG), which inhibits HIF hydroxylase, mediates the activation of hypoxic signaling and results in a significant increase in EV release (King et al., 2012). Another study revealed that hypoxia induced breast cancer cells to release more MVs than normoxia. This mechanism is mediated by HIF-dependent RAB22A gene expression, resulting in an increase in MV generation (Wang T. et al., 2014). In nasopharyngeal carcinoma, latent membrane protein 1 (LMP1) is the primary oncogene, as in Epstein-Barr virus (EBV) (Aga et al., 2014). LMP1 promotes the transformation and migration of epithelial cells. Aga et al. (2014) showed that LMP1 also increased the levels of HIF-1 in EVs. HIF-1 recovered from EVs is taken up by recipient cells and mediates the migration and invasion of nasopharyngeal carcinoma cells (Aga et al., 2014).

Hypoxia induced by tumor growth further limits cell proliferation. However, tumor cells in hypoxic areas produce EVs, which have several effects on the stroma. Additionally, EVs derived from hypoxic tumor cells influence important steps in cancer metastasis, such as angiogenesis. The HIF pathway is one of the most important pathways for tumor cells to survive in a hypoxic environment and mediates tumor angiogenesis in the TME. EVs containing HIF-1α can also induce tumor angiogenesis and metastasis.

## The Involvement of EVs Derived From Tumor Cells in Angiogenesis and Lymphangiogenesis in Endothelial Cells

Endothelial cells play essential roles in microvessel sprouting and angiogenesis associated with the TME and cancer metastasis. Angiogenesis is defined as the formation of new blood vessels from the existing vasculature to supply nutrients and oxygen to hypoxic organs (Maishi and Hida, 2017). VEGF is one of the key regulators of angiogenesis and promotes EC proliferation and migration and decreases EC apoptosis (Apte et al., 2019). The VEGF family includes VEGF-A, VEGF-B, VEGF-C, VEGF-D, VEGF-E, and PGF (Ferrara and Adamis, 2016). VEGF-A plays an important role in regulating angiogenesis during homeostasis and under disease conditions (Ferrara and Adamis, 2016). In a hypoxic TME, tumor cells and stromal cells secrete VEGF, leading to the survival of ECs and the formation of new blood vessels that may be structurally abnormal and leaky (Apte et al., 2019). It has been reported that tumor- and/or stroma-derived EVs promote angiogenesis related to VEGF. For example, VEGF on the surface of EVs derived from cancer cells promotes tumor angiogenesis (Ko et al., 2019). In glioma cells, EVs derived from U83-MG upregulate VEGF-A in HUVECs (Lang et al., 2017). These EVs contain the long non-coding (lnc) RNA-CCAT2, which is overexpressed in glioma tissues (Guo et al., 2016). The authors showed that EV-delivered lncRNA CCAT2 promoted angiogenesis by increasing the expression of VEGF-A in HUVECs (Lang et al., 2017). Colorectal cancer cell-derived EVs containing miR-25-3p target Krüppel-like factor 2 and regulate the expression of VEGF receptor 2, a VEGF receptor and the main signaling receptor for VEGF (Ferrara and Adamis, 2016); this results in vascular angiogenesis (Zeng et al., 2018). Thus, it has been well established that cancer cell-derived EVs directly transport VEGF or upregulate the VEGF pathway in ECs, resulting in tumor angiogenesis. In contrast, several reports have shown that various cancer cell-derived EVs regulate angiogenesis, EC proliferation and migration without stimulating VEGF expression. For example, EVs derived from breast cancer cells containing miR-9 effectively decreased suppressor of cytokine signaling 5 (SOCS5) expression in HUVECs. This decrease in SOCS5 led to EC migration by activating the JAK-STAT pathway (Zhuang et al., 2012). EV-delivered Wnt4 derived from colorectal cancer promotes angiogenesis through the Wnt/β-catenin signaling pathway in endothelial cells (Huang and Feng, 2017). Various types of tumor cells release EVs involved in the regulation of EC proliferation and migration through the VEGF- or non-VEGF-mediated pathway. The effects of tumor-derived EVs on ECs are summarized in Table 1.

**TABLE 1:** The effect of tumor-derived EVs on ECs.

Function	Cargo mediators	Origination	Mechanism	References
ECs migration and/or proliferation	VEGF	Several cancer cells	Upregulate VEGF pathway	Ko et al., 2019
	lncCCAT2	Glioma	Upregulate VEGF-A	Lang et al., 2017
	miR-25-3p	Colorectal cancer	Downregulate KLF2 leading to VEGFR2 upregulation	Zeng et al., 2018
	miR-9	Breast cancer	Downregulation SOCS5 leading to activated JAK-STAT pathway	Zhuang et al., 2012
	wnt4	Colorectal cancer	Increase β-catenin nuclear translocation leading to activated wnt/β-catenin pathway	Huang and Feng, 2017
Vascular permeability	miR-23a	Lung cancer	Suppress PHD-1,2 leading to accumulation of HIF-1α and downregulate ZO-1	Hsu et al., 2017
	miR-92-3p	Colorectal cancer	Downregulate Claudin 11	Yamada et al., 2019
	miR-103	HCC	Downregulate VE-cadherin, p120-catenin, and ZO-1	Choi and Moon, 2018
	miR-181c	Breast cancer	Downregulate PDPK1 leading to phosphorylated cofilin	Tominaga et al., 2015b
	miR-105	Breast cancer	Downregulate ZO-1	Zhou et al., 2014
lymphangiogenesis	LNMAT2	Bladder cancer	Upregulate PROX1	Chen et al., 2020
	miR-221-3p	Cervical cancer	Downregulate vasohibin-1 leading to activated ERK/AKT pathway	Zhou et al., 2019
EndoMT	miR-92-3p	Colorectal cancer	Upregulate snail and vimentin and downregulate Claudin 11	Yamada et al., 2019
	TGF-β	Melanoma	Induce TGF-β signaling	Yeon et al., 2018
ECM remodeling	mRNA MMP-2, 9	Renal cell carcinoma	Upregulate MMP-2, 9	Grange et al., 2011

*EC, endothelial cells; VEGF, vascular endothelial growth factor; lnc, long non-coding; KLF2, Krüppel-like factor; VEGFR2, vascular endothelial growth factor receptor 2; SOCS5; suppressor of cytokine signaling 5; PHD, prolyl hydroxylase; HIF, hypoxia-inducible factor; PDPK, 3-phosphoinositide-dependent protein kinase-1; LNMAT2, lymph node metastasis-associated transcript 2; PROX1, prospero homeobox 1; EndoMT, endothelial mesenchymal transition; TGF-β, transforming growth factor-β; ECM, extracellular matrix; MMP, matrix metalloproteinase.*

Tumor cells promote the loosening of intercellular adhesions between ECs by impairing adhesion molecules and gap junctions. Promotion of vascular permeability is related to enhanced cancer growth and metastasis by supplying enough nutrients and oxygen to growing tumors and promoting intravasation and extravasation. Accumulating evidence has revealed that tumor-associated EVs can modify vascular permeability (Figure 1). miR-23a in EVs is secreted by hypoxic lung cancer cells and suppresses prolyl hydroxylase (PHD) 1 and 2. PHD-1 and PHD-2 are regulators of HIF-1α (Berra et al., 2003). The suppression of PHD-1 and PHD-2 leads to the accumulation of HIF-1α in HUVECs (Hsu et al., 2017). miR-23a also downregulates ZO-1 expression in HUVECs and is related to tight junctions in the endothelium (Hsu et al., 2017). In addition, EV-delivered miR-92a-3p derived from colorectal cancer cells downregulates Claudin-11 (Yamada et al., 2019). Claudin-11 belongs to the claudin gene family and is one of the cell-to-cell adhesion molecules located at the tight junctions between ECs (Tsukita et al., 2019). VE-cadherin and p120-catenin are also adhesion molecules that contribute to endothelial junctions. Hepatoma cell-secreted EVs containing miRNA-103 impair endothelial junctions by directly inhibiting the expression of VE-cadherin, p120-catenin and ZO-1 (Choi and Moon, 2018). Thus, tumor-derived EVs influence vascular permeability and promote metastasis from the primary tumor.

An increase in vascular permeability in distant organs also promotes the extravasation of cancer cells and formation of the premetastatic niche. EV-delivered miR-181c from breast cancer cells downregulates 3-phosphoinositide-dependent protein kinase-1 (PDPK1) and disrupts the blood-brain barrier and consists of tight junctions between ECs and surrounding cells that contribute to low permeability and promote cancer metastasis in distant organs (Tominaga et al., 2015b). Another study showed that EV-delivered miR-105, which is a key regulator of ZO-1 and secreted by metastatic breast cancer cells, downregulated and destroyed tight junctions in endothelial monolayers. The same study also showed that treatment with anti-miR-105 suppressed brain metastasis *in vivo* (Zhou et al., 2014). ECs essentially have the ability of a physical barrier to separate blood from tissue and prevent tumor cell intravasation and extravasation. Tumor-derived EVs can lead to a disruption of adhesion molecules in ECs and promote not only cancer growth at the primary tumor but also the extravasation of tumor cells and formation of the premetastatic niche at distant organs. Tumor cells modify the structure of ECs to lose their physical barrier, resulting in tumor metastasis.

Tumor-derived EVs can also enhance tumor lymphangiogenesis and lymph node (LN) metastasis. It has been reported that different types of human cancer promote lymphangiogenesis, which is correlated with LN metastasis, and the presence of LN metastasis often reflects a poor prognosis (Karaman and Detmar, 2014). VEGF-C is a member of the VEGF family of angiogenic factors and is characterized as a lymphangiogenic growth factor (Su et al., 2007). Several studies have shown that VEGF-C promotes tumor-associated lymphangiogenesis and LN metastasis (Mandriota et al., 2001; Skobe et al., 2001). VEGFR-2 and VEGFR-3 are receptors for VEGF-C and VEGF-D. VEGFR-2 and VEGFR-3 also play important roles in the survival, proliferation and migration of lymphatic endothelial cells (LECs) (Joukov et al., 1996). There is no evidence that tumor-related EVs regulate lymphangiogenesis through the VEGF-C pathway. However, Fu et al. (2015) reported that miR-221-5p promoted the expression of VEGF-C in bladder cancer cells. Yang et al. (2017) reported that tumor-derived EVs contained miR-221, which contributed to tumor progression in glioma. miR-221-5p in tumor-derived EVs may induce lymphangiogenesis via the VEGF-C pathway.

Bladder cancer is one of the most commonly diagnosed urological malignancies (Bray et al., 2018). LN metastasis in patients with bladder cancer is related to a poor prognosis (Hautmann et al., 2012). Although VEGF-C plays an important role in lymphangiogenesis (Tacconi et al., 2015), LN metastasis in 20% of bladder cancers in a VEGF-C-independent manner (Chen et al., 2020). Chen et al. (2020) suggested that EV-related lymph node metastasis associated transcript 2 (LNMAT2) induced human LEC tube formation and migration *in vitro* and enhanced tumor lymphangiogenesis and LN metastasis *in vivo* through the upregulation of prospero homeobox 1 (PROX1). Another study showed that cervical squamous cell carcinoma-derived EVs promoted LEC migration and tube formation *in vitro* and facilitated lymphangiogenesis and LN metastasis *in vivo* (Zhou et al., 2019). These EVs contained miR-221-3p, which downregulated vasohibin-1 expression in LECs. This miR-221-3p-vasohibin-1 axis activated the AKT/ERK signaling pathway, resulting in lymphangiogenesis without VEGF-C (Zhou et al., 2019). These mechanisms may explain why tumor-derived EVs induce tumor lymphagenesis and LN metastasis in a VEGF-C-independent manner. There is little evidence of tumor- or stromal cell-derived EVs associated with lymphangiogenesis and LN metastasis.

Tumor cells may regulate lymphangiogenesis through the EV-mediated VEGF-C or non-VEGF-C pathway. If tumor lymphangiogenesis-mediated tumor-related EVs are elucidated, we can predict LN metastasis in various tumor cell types by sampling various biofluids and prevent LN metastasis by inhibiting lymphangiogenesis-mediated EV secretion.

## The Contributions of EVs Derived From Tumor-Associated Endothelial Cells to Angiogenesis

Numerous studies have shown that tumor-derived EVs influence ECs to promote vascular permeability, angiogenesis and metastasis. On the other hand, ECs and tumor-associated ECs (TECs) also secrete EVs, and several studies have suggested that these EVs contribute to angiogenesis and metastasis. ECs in a hypoxic environment secrete EVs, which include lysyl oxidase-like 2 (LOXL2) (de Jong et al., 2016). LOXL2 is a member of the LOX family and regulates extracellular matrix (ECM) remodeling, angiogenesis, and premetastatic niche formation. EV-associated LOXL2 derived from TECs in a hypoxic environment induces angiogenesis. Another study showed that breast and ovarian cancer tissue released IL-3, which influenced ECs in the TME. IL-3-treated ECs secrete EVs, serving as a paracrine mechanism for neighboring ECs (Lombardo et al., 2018). When TECs were treated with an anti-IL-3Rα blocking antibody, EV-delivered miR-214-3p was upregulated, and miR-24-3p was downregulated. These miRNAs regulate neovessel formation through the Wnt/β-catenin pathway (Table 2). Anti-IL-3R antibody-treated EVs derived from TECs regressed tumor-treated EC-induced neovessels *in vivo* (Lombardo et al., 2018). In addition, TECs also contribute to immune suppression via EVs. Lopatina et al. (2020) showed that EVs collected from ECs associated to head and neck squamous cell carcinoma were able to increase IL-6 and TGF-β in peripheral blood mononuclear cells (PBMC) and increase the T regulatory cell population. TEC-EVs also increased the expression of MALAT1 in PBMC resulting in M2-type differentiation of macrophages. These studies indicate that EVs derived from ECs in the TME carry several factors, resulting in tumor angiogenesis and immune suppression (Figure 1).

**TABLE 2:** The function of tumor cells and stromal cells-derived EVs.

Function	Cargo mediators	Origination	Target	Mechanism	References
ECs migration and/or proliferation	miR-24-3p miR-214-3p	TECs	ECs	Induce wnt/β-catenin pathway	Lombardo et al., 2018
Differentiation into CAFs	several miRNAs	Gastric cancer	Fibroblasts	Induce CAFs to secrete CXCL1, 8	Naito et al., 2019
	TGF-β	Prostate cancer	MSC	Upregulate α-SMA in MSC	Chowdhury et al., 2015
	TGF-β	Bladder cancer	Fibroblasts	Induce Smad pathway in fbroblasts	Ringuette Goulet et al., 2018
	miR-125	Breast cancer	Fibroblasts	Upregulate α-SMA in fibroblasts	Vu et al., 2019
	miR-1247-3p	HCC	Fibroblasts	Upregulate B4GALT3 leading to activated β1-integrin-NF-κB pathway in fibroblasts	Fang et al., 2018
ECM remodeling	LOXL2	EC	none	Directly ECM remodeling	de Jong et al., 2016
	mRNA MMP-1	Ovarian cancer	mesothelial cells	Upregulate MMP-1 in mesothelial cells	Yokoi et al., 2017
	mRNA MMP-9	Platelets	ECs	Upregulate MMP-9 in ECs	Janowska-Wieczorek et al., 2005
	ADAM 10	CAFs	several cancer cells	Induce RhoA-Notch signaling	Shimoda et al., 2014
	miR-139	CAFs	Gastric cancer	Suppress MMP-11 in gastric cancer	Xu et al., 2019
	Unknown	CAFs	Gastric cancer	Upregulate MMP-2 in gastric cancer	Miki et al., 2018
Coagulability	TF	Ovarian cancer		Hypercoagulability	Koizume et al., 2016
	TF	Platelets		Hypercoagulability	Freyssinet and Toti, 2010
VM formation	VEGF	TECs	HCC	Upregulate VEGF	Zeng et al., 2019

*EC, endothelial cell; TEC, tumor associated endothelial cell; CAF, cancer associated fibroblast; CXCL, chemokine (C-X-C motif) ligand; TGF-β, transforming growth factor-β; SMA, smooth muscle actin; MSC, mesenchymal stem cell; HCC, hepatocellular carcinoma; B4GALT3, β-1,4-galactosyltransferases III; ECM, extracellular matrix; LOXL2, lysyl oxidase-like 2; MMP, matrix metalloproteinase; ADAM, a disintegrin and metalloproteinase; TF, tissue factor; VM, vasculogenic mimicry; VEGF, vascular endothelial growth factor.*

Data from several clinical studies have suggested that EV-derived ECs are involved in cancer prognosis. In patients with lung cancer, circulating levels of endothelial-derived EVs, especially MVs, are independent predictors of mortality (Wang C.C et al., 2014). In breast cancer patients treated by chemotherapy, the decrease in endothelial-derived EV (defined as CD144-positive MV) levels after chemotherapy is associated with prolonged overall and disease-free survival (García Garre et al., 2018). Although tumor cells can educate ECs (TECs) and promote angiogenesis in neighboring ECs, the mechanism by which TEC-associated EVs contribute to tumor angiogenesis and metastasis remains unclear. However, these previous studies indicate that crosstalk between tumor cells and ECs via EVs mediates tumor progression and angiogenesis.

## Tumor-Derived EVs Promote Endothelial to Mesenchymal Transition (EndoMT)

Epithelial cells in tumors can change their phenotype, downregulate epithelial characteristics and acquire mesenchymal characteristics (Lamouille et al., 2014). This cellular program is called epithelial-mesenchymal transition (EMT). EMT is an important step in the malignant progression of almost all types of carcinoma (Dongre and Weinberg, 2019). When EMT occurs, cell-to-cell tight junctions and adhesive molecules in epithelial cells are lost, and cancer cells acquire migration ability (Quintero-Fabián et al., 2019).

Endothelial cells in the TME can also modify their endothelial phenotype switch to the mesenchymal profile in a manner similar to that of epithelial cells. This phenotype switch in ECs is referred to as endothelial-to-mesenchymal transition (EndoMT) (Platel et al., 2019). Recently, EndoMT has been described as an important process in the development and dissemination of cancer. In a way similar to that during EMT, ECs induced to switch to the mesenchymal phenotype lose capillaries and endothelial markers such as CD31, Tie-2 and VE-cadherin, acquire mesenchymal markers such as N-cadherin, fibroblast-specific protein-1, vimentin, α-SMA, and type I/III collagen and become more invasive, with increased migratory abilities (Nie et al., 2014; Platel et al., 2019). Although various molecules, such as interleukin-1β (IL-1β) and TNF-α, can promote EndoMT, transforming growth factor-β (TGF-β) plays a major role in EC conversion during EndoMT (Medici et al., 2011; Kovacic et al., 2012; Sanchez-Duffhues et al., 2018). TGF-β stimulates the Smad signaling pathway, which regulates the differentiation transcription factors Snail, Twist, Slug, and ZEB (Miyazono, 2000; Dongre and Weinberg, 2019). These transcriptional factors inhibit the expression of genes related to the epithelial state and promote the expression of genes related to the mesenchymal state (Dongre and Weinberg, 2019). In addition, Snail and ZEB2 activate the expression of matrix metalloproteinase (MMP), which promotes degradation of the basement membrane, causing vascular invasion in the primary tumor (Miyoshi et al., 2004, 2005). EndoMT functions to promote the extravasation of tumor cells by increasing endothelial vascular permeability and causing tumor metastasis. A recent study showed that EVs containing miR-92a-3p derived from colon cancer cells upregulated mesenchymal markers such as snail and vimentin and downregulated the tight junction marker ZO-1 in HUVECs. This biological process is referred to as “partial-EndoMT” (Yamada et al., 2019). This process promotes angiogenesis by increasing proliferation, motility, and tube formation in HUVECs. In addition, TGF-β on the surface of melanoma-derived EVs trigger the phenotypic switch from an endothelial phenotype to a mesenchymal phenotype concerning gene expression (Yeon et al., 2018). These phenomena transdifferentiate ECs into cancer-associated fibroblasts (CAFs) (Togo et al., 2013). CAFs are the most abundant cells in the TME, secrete several proangiogenic signaling factors and play an important role in metastasis and angiogenesis (Wang et al., 2019).

The phenotypic switch in ECs via tumor-related EVs is thought to contribute to the intravasation and extravasation of tumor cells via an impairment in vascular permeability and becomes a resource for CAFs (Figure 1). EndoMT also promotes the proliferation and migration of ECs (i.e., mesenchymal abilities), resulting in tumor angiogenesis. These findings suggest that ECs that undergo the phenotypic switching migrate to distant organs via tumor-derived EVs and induce tumor angiogenesis in the premetastatic niche as well as in the primary tumor.

## The Function of EVs Associated With Cancer-Associated Fibroblasts and Tumor Cells in Tumor Angiogenesis

Cancer-associated fibroblasts are the main component of stromal cells in the TME. They originate from various types of cells, such as epithelial cells, resident fibroblasts, bone marrow derived from mesenchymal stem cells, hematopoietic stem cells, and ECs (Quante et al., 2011; Mitra et al., 2012). Myofibroblasts are one of the main subtypes of CAFs that express α-smooth muscle actin (α-SMA) (Sappino et al., 1988; Kraman et al., 2010). Because there are various subtypes of CAFs, it is not clear what markers are specific for CAFs. However, α-SMA is currently the most commonly used specific marker for CAFs. CAFs have several important functions in tumor angiogenesis. They secrete various proinflammatory cytokines that promote angiogenesis and vasculogenesis, such as IL-1β, IL-6, and IL-8, and recruit inflammatory cells, such as macrophages (Erez et al., 2010). MMPs secreted by CAFs degrade the extracellular matrix and basal membrane and lead to tumor angiogenesis (Erez et al., 2010). Several proangiogenic signaling factors derived from CAFs, such as VEGF, TGF-β, hepatocyte growth factor (HGF), and SDF-1, also promote angiogenesis and vasculogenesis (Orimo et al., 2005; Chowdhury et al., 2015). PDGF-β and basic fibroblast growth factor (bFGF) can be secreted by cancer cells to activate CAFs indirectly to promote tumor angiogenesis (Shao et al., 2000; Strutz et al., 2000).

Evidence of the mechanism by which stromal cells are activated by tumor cells via EVs is gradually accumulating (Table 2). According to a recent study, a highly metastatic type of diffuse-type gastric cancer cell-derived EVs contain several miRNAs (Naito et al., 2019). CAFs take up these EVs, which effect CAFs, to secrete chemokine (C-X-C motif) ligand (CXCL) 1 and CXCL8 via miR-155, miR-193b, and miR-210. Another study suggested that cancer cell-derived EVs promote the phenotypic switch of cancer stromal cells into CAFs. For example, EVs containing TGF-β secreted by prostate cancer cells induced mesenchymal stem cell differentiation into CAFs that exhibited α-SMA-positive myofibroblast cells (Chowdhury et al., 2015). In addition, bladder cancer cell-derived EVs contain TGF-β. These EVs induced normal fibroblasts into CAFs through the Smad pathway (Ringuette Goulet et al., 2018). Moreover, EVs derived from breast cancer cells are taken up by cancer stromal cells at a high rate. These EVs contain miR-125, which is transferred to normal fibroblasts and upregulates α-SMA (Vu et al., 2019). EndoMT can also be the source of CAFs via cancer cell-derived EVs, as mentioned above. In primary tumors, tumor-derived EVs promote the conversion of several types of cells into CAFs, and CAFs secrete various types of proangiogenic factors, resulting in tumor angiogenesis.

Cancer cell-derived EVs contribute to the conversion of fibroblasts into CAFs in the premetastatic niche. In HCC, miR-1247-3p is carried to the premetastatic niche in the lung through EVs and converts fibroblasts to CAFs by decreasing the expression of its target, β-1,4-galactosyltransferase III (B4GALT3), to activate the β1-integrin-NF-κB pathway (Fang et al., 2018). Integrins promote the nuclear translocation of NF-κB, and the NF-κB signaling pathway promotes the growth and migration of epithelial cells (Nikolopoulos et al., 2005). B4GALT3 also inhibits β1-integrin activation and stability (Liao et al., 2015). Serum EVs containing miR-1247-3p are elevated in HCC patients with lung metastasis compared with healthy individuals or HCC patients without lung metastasis (Fang et al., 2018). The NF-κB pathway mediates various proangiogenic factors, such as IL-1, IL-8, tumor necrosis factor, IL-6, VEGF, MMP-2, and MMP-9 (Maeda and Omata, 2008). This study indicates that cancer cell-derived EVs induce angiogenesis in the premetastatic niche through the NF-κB pathway.

Not only cancer cells but also CAFs secrete EVs, which may stimulate cancer metastasis and angiogenesis. Miki et al. (2018) revealed that CD9-positive EVs from CAFs were taken into scirrhous-type gastric cancer cells. MMP-2 expression in scirrhous-type gastric cancer cells was significantly decreased by CD9-siRNA. CD9 positivity was significantly related to LN metastasis and venous invasion. MMP-2 is well known as a modulator of dynamic remodeling of the ECM (Quintero-Fabián et al., 2019). MMP-2 correlates with neocapillary network growth, HUVEC migration and angiogenesis. CAF-derived EVs may induce angiogenesis and lymphangiogenesis through MMP-2. Crosstalk between cancer cells and CAFs plays an important role in angiogenesis. Recent studies have revealed that cancer cell-derived EVs might be associated with converting stromal cells into CAFs, which upregulate angiogenesis in primary tumors and distant organs. Evidence of the mechanisms of interaction between tumor cells and CAFs via EVs has accumulated. CAFs constitute the majority of cells in the TME and are thought to secrete various angiogenic factors, including EVs.

## The Roles of Matrix Remodeling and Angiogenesis Associated With EVs

The ECM consists of collagen, proteoglycans, elastin, fibronectin and integrins that provide structural and biochemical support for cancer cell growth (De Palma et al., 2017; Nawaz et al., 2018). The ECM is versatile and continues to be remodeled. Tumor cells, CAFs, ECs and other stromal cells are associated with matrix remodeling in the primary tumor microenvironment (Shuman Moss et al., 2012; Eble and Niland, 2019). Tumor cells invade the ECM as a barrier and result in cancer cell migration. Moreover, they remodel the ECM in distant organs to facilitate formation of the premetastatic niche. Angiogenesis also requires a disruption in endothelial-lined vessels through the sprouting of ECs (Quintero-Fabián et al., 2019). Sprouting angiogenesis is related to the degradation of intercellular relationships, cell-matrix adhesion molecules, the ECM, and basement membrane components by MMPs, plasmin and other proteases (De Palma et al., 2017; Quintero-Fabián et al., 2019). Thus, it is important to understand ECM remodeling in the TME to elucidate cancer metastasis and angiogenesis.

**FIGURE 2:**
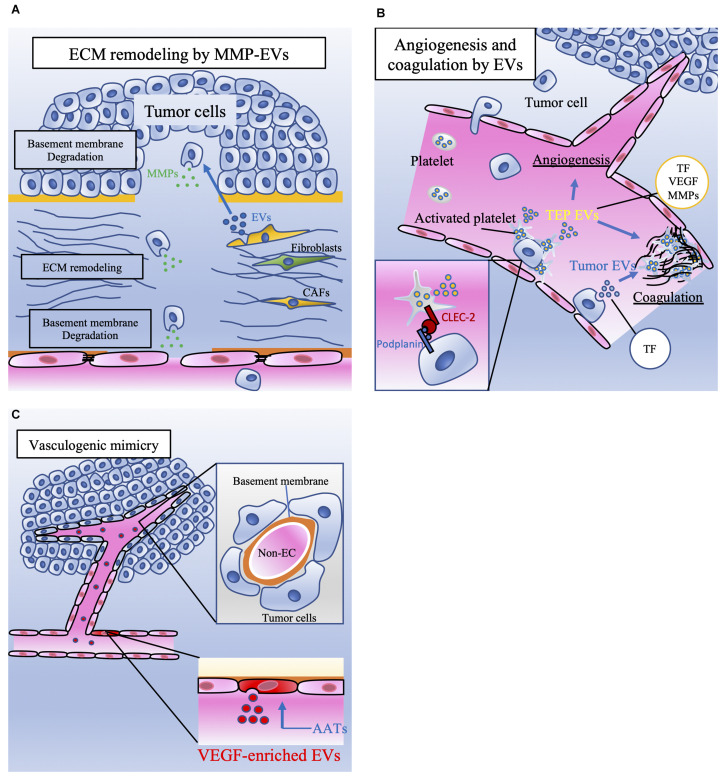
The involvement of EVs released by tumor cells and stromal cells in tumor metastasis. **(A)** Extracellular matrix (ECM) remodeling is mediated by tumor cells and cancer-associated fibroblasts (CAFs). Extracellular vesicles (EVs) contain matrix metalloproteinases (MMPs), which degrade the basement membrane. ECM remodeling induces tumor cell migration and vascular sprouting, resulting in tumor progression. **(B)** Platelet microparticles (PMPs) are the most abundant EVs in the blood and are secreted by activated platelets. Platelets can be activated by tumor cells. Podoplanin is expressed on the surface of tumor cells, and C-type lectin-like receptor 2 (CLEC-2) is expressed on the surface of platelets. The interaction between CLEC-2 and podoplanin activates platelets referred to as tumor-educated platelets (TEPs). TEPs play important roles in angiogenesis and coagulation through the secretion of these EVs or PMPs containing tissue factor (TF), vascular endothelial growth factor (VEGF), and matrix metalloproteinases (MMPs). Tumor cells also directly secrete procoagulant EVs that contain TF. TF activates plasmatic coagulation cascades and induces platelet activation. **(C)** Although anti-angiogenic targeted therapy (AAT) is a key therapy for malignant tumors, the effect of AATs is transient. One of the mechanisms of AAT resistance is vasculogenic mimicry (VM). VM consists of tumor cells that have capillary-like structures and basement membranes but not endothelial cells. ECs treated with AATs secrete vascular endothelial growth factor (VEGF)-enriched EVs. These EVs enhance VM, which supplies nutrients and oxygen to tumor cells and contributes to tumor progression.

Recently, relevant studies in which tumor-derived EVs containing MMPs were associated with angiogenesis have gradually accumulated (Table 2). The involvement of MMPs in tumor angiogenesis is highly important and well known. During the angiogenesis process, ECs secrete MMPs to remodel the basement membrane (De Palma et al., 2017). MMPs are a family of 23 zinc-dependent endopeptidases that degrade ECM components and the basement membrane and are related to tumor cell migration into adjacent tissue. Not only ECs but also several types of cells, such as cancer cells, CAFs, and platelets, secrete EVs containing MMPs and result in angiogenesis. For example, EVs derived from CD105-positive cells in renal cell carcinoma trigger angiogenesis and promote formation of the premetastatic niche (Grange et al., 2011). These EVs contain several mRNAs, such as VEGF, FGF, MMP-2, and MMP-9. In addition, EVs derived from highly metastatic ovarian cancer cells contain MMP-1 mRNA, resulting in destruction of the peritoneal mesothelium barrier (Yokoi et al., 2017). Platelets activated by tumor cells also secrete MVs that stimulate the mRNA expression of MMP-9, VEGF, and IL-8 in HUVECs (Janowska-Wieczorek et al., 2005). CAFs secrete ADAM10-rich EVs, which promote the RhoA and Notch signaling pathways in cancer cells, and play an essential role in enhancing cell motility (Shimoda et al., 2014). Based on the findings described above, cancer cells and cancer stromal cells can create favorable conditions for growth through ECM remodeling and promote tumor angiogenesis (Figure 2A).

However, cancer stromal cells may suppress cancer progression. Xu et al. (2019) suggested that EVs containing miR-139 derived from CAFs in gastric cancer suppressed MMP-11 secretion via EVs derived from CAFs. They also showed that miR-139 in EVs inhibited tumor growth and metastasis *in vitro* and *in vivo* (Xu et al., 2019). CAFs may contribute to cancer progression and/or suppression through EVs, but the exact mechanism is unclear.

## The Link Between Cancer Cells and Platelets in EVs

Platelets contribute to pathological conditions such as inflammation, coagulation and tumor progression. The expression of podoplanin on cancer cells is one of the representative mechanisms of platelet activation by cancer cells (Haemmerle et al., 2018). C-type lectin-like receptor 2 (CLEC-2) on the surface of platelets can bind to podoplanin. After an interaction between CLEC-2 and podoplanin activates platelets, activated platelets promote hypercoagulation, inflammation, tumor growth, and metastasis (Takagi et al., 2013). Activated platelets, which are referred to as tumor-educated platelets (TEPs), were discovered in the 19th century (In ’t Veld and Wurdinger, 2019). Platelet microparticles (PMPs) are a predominant population of not only platelet-derived EVs but also all EVs in the blood, accounting for approximately 70–90% (Zmigrodzka et al., 2016). PMPs secreted by activated platelets enhance angiogenesis, vascular permeability, and coagulation (Wojtukiewicz et al., 2017; Haemmerle et al., 2018). In the 1860s, Armand Trousseau observed that an increase in venous thrombosis and/or blood hypercoagulability was associated with certain cancers; this is now referred to as “Trousseau’s syndrome” (Trousseau, 1865). Trousseau’s syndrome causes venous thromboembolism (VTE), which includes deep venous thrombosis and pulmonary embolism (Campello et al., 2011). PMPs are involved in coagulation as a source of tissue factor (TF) and phosphatidylserine (Freyssinet and Toti, 2010). High PMP levels are associated with aggressive tumors and thrombotic events. It has been reported that EVs containing TF from aggressive breast cancer cell lines exhibit more platelet aggregation activity than those from less invasive breast cancer cell lines and are associated with an increased risk of thrombosis (Gomes et al., 2017), and the procoagulant activity is of PMPs in cancer patients with VTE is high at baseline, suggesting that this activity can be used to predict VTE (van Doormaal et al., 2012). Not only platelets but also tumor cells secrete EVs and have procoagulant ability. Koizume et al. (2016) showed that ovarian clear-cell carcinoma (CCC), a subtype of ovarian cancer, secreted higher levels of TF within EVs than breast cancer. These EVs contributed to the increased plasma coagulability of CCC tumor-bearing mice. A hypoxic environment enhances CCC to secrete EVs enriched with TF. Although TEPs play an important role in tumor hypercoagulability, tumor cells also directly secrete procoagulant EVs and promote VTE. TF activates plasmatic coagulation cascades and induces platelet activation (Han et al., 2014). Platelet activation by tumor cells induces tumor invasion, immune evasion, vascular arrest, and extravasation (Schlesinger, 2018). Thus, hypercoagulability induced by TEPs and tumor cells contributes to tumor progression.

Several studies have suggested that PMPs are associated with not only VTE but also cancer angiogenesis and patient prognosis (Figure 2B). PMPs in lung cancer cells can induce the mRNA expression of MMP-9, VEGF, IL-8, and HGF in HUVECs (Janowska-Wieczorek et al., 2005), mediate angiogenic functions by VEGF, PDGF, TGF-β, and bFGF (Haemmerle et al., 2018), and stimulate the proliferation, chemotaxis and tube formation of HUVECs (Kim et al., 2004). In addition, these contents of PMPs are predictors of cancer metastasis in gastric cancer (Kim et al., 2003). In patients with prostate cancer, a significant relationship between high levels of PMPs and poor survival rates was found (Helley et al., 2009). TEPs are thought to promote angiogenesis and be associated with the prognosis of patients with cancer. Therefore, tumor cells utilize platelets to create a beneficial microenvironment for themselves. TEPs contribute to several important steps in tumor metastasis. Although antiplatelet therapy can be used as anticancer therapy, there is no reliable clinical evidence for antiplatelet therapy for tumor metastasis (Xu et al., 2018). Clinical evidence, such as that from a large randomized control trial, is needed before antiplatelet therapy can be translated into anticancer therapy.

## The Mechanism of Overcoming Anti-Angiogenic Targeted Therapy: Vasculogenic Mimicry

Anti-angiogenic targeted therapy (AAT) is a key therapy for many tumor types and a well-established research area (Reck et al., 2018). AATs help starve tumors by suppressing tumor angiogenesis and destroying preexisting tumor-associated blood vessels (Zeng et al., 2019). As mentioned above, VEGF and its receptors play an important role in EC survival and tumor angiogenesis. Bevacizumab is one of the first commercially used AATs and a humanized monoclonal antibody against VEGF-165 (Pinto et al., 2016). Currently, several AAT drugs can be used and have been well established. However, the effect of AATs is transient. Tumor cells can escape angiogenesis inhibition through different forms of neovasculature and acquire resistance to AATs (Pinto et al., 2016). These processes may result in limited outcomes in patients with aggressive tumors and lead to cancer-related death.

One of the mechanisms by which AATs function is vascular mimicry (VM) (Maniotis et al., 1999; Folberg et al., 2000). In 1999, it was described that the formation of tubular structures was consistent not with that of ECs but of cancer cells within melanomas (Valdivia et al., 2019). Thereafter, the concept of VM has been reported in various types of solid tumors until now and was significantly associated with cancer differentiation, LN metastasis (Sun B. et al., 2017; Hujanen et al., 2020), distant organ metastasis, and poor prognosis (Yang et al., 2016). VM is described as tumor cells acquiring the endothelial-like capillary form and providing oxygen and nutrients to hypoxic tumor cells (Maniotis et al., 1999; Folberg et al., 2000). The basal membrane consists not only of blood and lymph vessels but also a variety of glycoproteins that are stained by periodic acid-Schiff (PAS) (Folberg and Maniotis, 2004). However, one characteristic of VM is the absence of CD31 and CD34, which are defined as endothelial markers (Seftor et al., 2001). VM is defined as PAS positive and CD31 or CD34 negative (Angara et al., 2017; Valdivia et al., 2019; Hujanen et al., 2020).

Vascular mimicry can be induced by a hypoxic environment. Several studies have suggested that HIF-1α is involved in VM (Serova et al., 2016). The expression of HIF-1α, MMP-2, VE-cadherin, and Twist-1 in triple-negative breast cancer cells is higher than that in non-triple-negative breast cancer cells (Sun H. et al., 2017). HIF-1α activation is correlated with EBV-associated epithelial cancers, such as nasopharyngeal carcinoma and gastric cancer (Xiang et al., 2018). On the other hand, VM associated with VEGF is controversial. Some reports suggested that anti-VEGF agents limited VM (Xiang et al., 2018). Another report suggested that the suppression of Flk-1 (VEGFR-2) activity and gene expression inhibited VM *in vivo* (Scully et al., 2012). Further investigation is needed to understand the mechanism underlying the relationship between VM and signaling pathways.

On the other hand, Zeng et al. (2019) showed that EVs derived from TECs might underlie the mechanism of resistance to anti-VEGF therapy (Table 2). miR-9 induces angiogenesis through the VEGF signaling pathway and promotes autophagy (Fu et al., 2015). The authors transfected HUVECs with miR-9 to mimic TECs in HCC. When vandetanib, an angiogenesis inhibitor, was administered to TECs, miR-9-induced angiogenesis and autophagy were inhibited. Instead, TECs secreted a high level of VEGF-enriched EVs. These EVs induced vascular network formation *in vitro* and enhanced endothelial vasculogenesis and VM *in vivo* (Zeng et al., 2019; Figure 2C). This mechanism may be one of the accountable phenomena by which tumor cells acquire resistance to AATs. Although there are few studies on the involvement of EVs in VM, tumor-associated EVs are thought to be related to VM, leading to resistance to AATs and tumor progression. A clinical study suggested that VM was related to tumor progression. Hujanen et al. (2020) showed in a meta-analysis that vascular mimicry positivity was strongly associated with poor overall survival in patients with squamous cell carcinoma of the head and neck or esophagus.

These studies indicate that EVs in the TME are implicated in VM after AATs, resulting in AAT resistance. If VM can be prevented, AATs will be beneficial to patients with cancer. The prevention of EV release in TECs induced by AATs can be one of the target therapies for VM and AAT resistance. Another study showed that miR-124 inhibit VM formation of cervical cancer through inhibition of MMP-2, MMP-9, and VEGF (Wan et al., 2014). miR-9 also inhibits VM formation by regulating stathmin expression in glioma cells (Song et al., 2013). These studies are not associated with EVs. However, these miRNAs are delivered by EVs in several cancer cells (Sueta et al., 2017; Lu et al., 2018). EVs loading these miRNAs can be also therapeutic candidates for VM formation and AAT resistance.

## Concluding Remarks

A hypoxic environment due to the rapid and uncontrollable growth of tumor cells influences tumor cells and stromal cells to secrete not only several chemical mediators but also EVs, which contribute to neovasculature and metastasis. Neovasculature is one of the most important steps in metastasis and components of the TME. Tumor and stromal cells communicate with each other via EVs in the TME. Tumor-derived EVs induce angiogenesis in ECs in a VEGF-dependent or VEGF-independent manner. EVs secreted by tumor cells impair EC adhesion molecules and gap junctions to promote vascular permeability. The loss of endothelial markers and the acquisition of mesenchymal markers is referred to as EndoMT, which is activated by tumor-related EVs, contributes to the intravasation and extravasation of tumor cells and becomes a resource for CAFs. CAFs are derived from various types of cells and secrete EVs related to angiogenesis. Several types of cells, such as ECs, CAFs, platelets, and tumor cells, secrete EVs containing MMPs and result in ECM remodeling and angiogenesis. Platelets educated by tumor cells secrete PMPs that induce inflammation and coagulation. PMPs are the most abundant EVs in the blood and are associated with tumor angiogenesis, VTE, and patient prognosis. AAT is an important therapy for tumor progression. However, tumor cells induced by AATs establish capillary forms without vascular-related cells; this is referred to as VM.

As mentioned in this review article, we provide evidence that tumor and stromal cells are involved in tumor neovasculature through crosstalk with each other via EVs. A number of studies have suggested that tumor cells rearrange surrounding cells and the ECM using EVs, and these studies have helped understand the mechanism of tumor angiogenesis. In addition, recent studies on the involvement of non-malignant cells in cancer progression and regression have gradually accumulated.

The function of EVs derived from tumor cells and stromal cells may shed light on the mechanisms of cancer angiogenesis and progression. Tumor-derived EVs may also contribute to tumor cells becoming resistant to anti-tumor therapy, such as VM induced by AATs. Therefore, if we discover a method to prevent the release of EVs associated with tumor angiogenesis, lymphangiogenesis, and VM, it will be a candidate as effective treatment methods to improve not only cancer angiogenesis and metastasis but also cancer prognosis. In fact, accumulating evidence for therapeutic candidates associated with EVs indicate that several biomolecules in EVs have significant advantages for cancer diagnosis and prognosis (Wang et al., 2018). In addition, EVs can be loaded with several molecules such as chemotherapy agents (Agrawal et al., 2017) and nucleic acids including mRNA, miRNA, and interfering RNA (Jiang et al., 2017). Although further investigation is needed to utilize these techniques for tumor therapy, the approaches to EVs will provide a step forward in the overcoming of cancer.

## Author Contributions

NK, YY, SK, NA, and TO developed the concept of the review. NK, YY, and TO drafted the review. SK and NA corrected and reviewed the review. All authors contributed to the article and approved the submitted version.

## Conflict of Interest

The authors declare that the research was conducted in the absence of any commercial or financial relationships that could be construed as a potential conflict of interest.
